# Advances in COVID-19 research until November 2020: Update from the UNCOVER registry

**DOI:** 10.7189/jogh.11.03022

**Published:** 2021-01-30

**Authors:** Xiaomeng Zhang, Wei Xu, Marshall Dozier, Farirai P Nzvere, Prerna Krishan, Yazhou He, Xue Li, Evropi Theodoratou

**Affiliations:** 1 Centre for Global Health, Usher Institute, University of Edinburgh, Edinburgh, UK; 2 Information Services, University of Edinburgh, Edinburgh, UK; 3 West China School of Public Health and West China Fourth Hospital, Sichuan University, Chengdu, PR China; 4 School of Public Health, Zhejiang University, Hangzhou, PR China; 5 Second Affiliated Hospital, Zhejiang University, Hangzhou, PR China; 6 Cancer Research UK Edinburgh Centre, Medical Research Council Institute of Genetics and Molecular Medicine, University of Edinburgh, Edinburgh, UK

Since the declaration of Coronavirus Disease 2019 (COVID-19) pandemic on 11 March 2020 by the WHO, an accumulated number of at least 70 461 926 confirmed cases and 1 599 704 deaths in 216 countries/territories has been recorded by 14/12/2020 [1]. In line with the established WHO global research roadmap for COVID-19 [2], the UNCOVER (Usher Network for COVID-19 Evidence Reviews) group committed to summarise COVID-19 evidence-based reviews for policymakers, clinicians and researchers to respond quickly to the outbreak of COVID-19. To assist in policy and clinical decision making and to avoid duplicate efforts, we created the UNCOVER registry of reviews, an online collection of published and ongoing reviews. Details of the UNCOVER registry have been previously reported [3] and here we present an update in relation to the main characteristics of the current collection.

**Figure 1:**
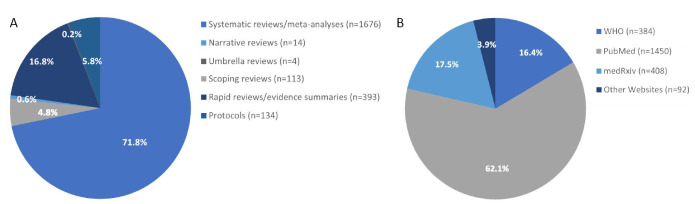
Review types and review sources of the included reviews in the UNCOVER registry. **Panel A.** Review types. **Panel B.** Review sources. medRxiv preprints were replaced with the peer-reviewed publications once they have been published in peer-reviewed journals; the review types displayed directly in the searching results of the website and the review sources can be accessed through the filter.

As of 16/11/2020, the UNCOVER registry includes 2334 different types of published or ongoing reviews (https://www.ed.ac.uk/usher/uncover/register-of-reviews), including 1676 systematic reviews and meta-analyses, 393 rapid reviews, 113 scoping reviews, four umbrella reviews, 14 narrative reviews and 134 protocols (**Figure 1**, Panel A) [4]. The website has been accessed a total of 9094 times with 4169 times abstract viewing and 3150 times completed review viewing. The publication sources for COVID-19 evidence-based reviews in the UNCOVER register include PubMed (n = 1450), WHO COVID-19 database (n = 384), medRxiv (n = 408), and other websites (n = 92) such as the national collaborating centre for methods and tools [5] and The Centre for Evidence-Based Medicine [6] (**Figure 1**, Panel B).

**Figure 2:**
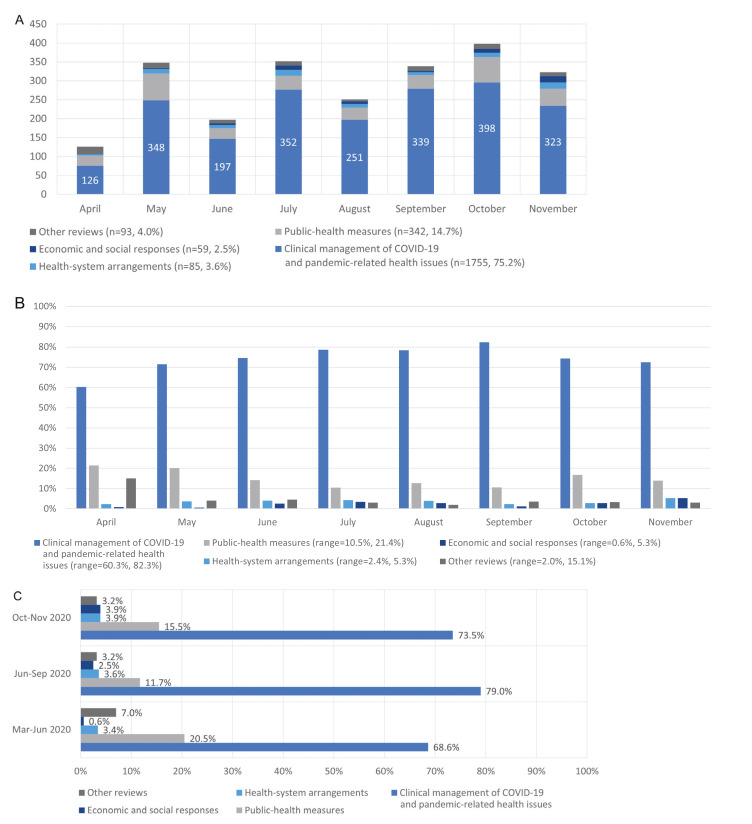
Number of reviews and distribution of review topics of the UNCOVER registry over time **Panel A.** Number of reviews entered in UNCOVER registry by the month of entry. **Panel B.** Proportion of review topics entered in the UNCOVER registry by the month of entry. **Panel C.** Proportion of review topics entered in the UNCOVER registry by the three time periods.

Research topics of indexed reviews were categorised thematically according to McMaster University COVIDEND (COVID-19 Evidence Network to support Decision-making) taxonomy into five groups: 1) Clinical management of COVID-19 and pandemic-related health issues (n = 1755, 75.2%); 2) Public-health measures (n = 342, 14.7%); 3) Health-system arrangements (n = 85, 3.6%); 4) Economic and social responses (n = 59, 2.5%); 5) Other reviews (n = 93, 4.0%) (**Figure 2**, Panel A) [7]. We further categorised each review into 22 subgroups under the five COVIDEND groups (**Table 1**):

**Table 1:** Categories and selected sub-categories of research topics of reviews included in the UNCOVER registry.

Clinical management of COVID-19 and pandemic-related health issues (n = 1755)	Public-health measures (n = 342)	Health-system arrangements(n = 85)	Economic and social responses (n = 59)	Other reviews(n = 93)
**Clinical features (n = 372)**	**Infection (n = 37)**	**Health care resource arrangement (n = 38)**	**Education (n = 11)**	**Comparison of COVID-19, SARS, and MERS (n = 32)**
• Symptoms	• Infection route	• Allocation of ICU beds and ventilators	• Public health education	• Viral load dynamics
• Biochemical indicators	• Secondary infection	• Primary health care	**Social consequences (n = 29)**	• Duration of viral shedding
**Clinical tests (n = 147)**	**Transmission (n = 115)**	• Orthodontic care	• Bereavement	**Published articles (n = 25)**
• Effectiveness of diagnostic test	• Transmission route	• Neurosurgical practice	• Domestic violence	• Methodological quality of COVID-19 systematic reviews
• CT imaging and findings	• R number	**Clinical departments arrangement (n = 42)**	**Economy (n = 7)**	**Coronavirus genomic RNA packaging (n = 19)**
• Lung ultrasound	• Incubation	• Hospital surge capacity planning	**Governance (n = 6)**	**Others (n = 17)**
**Clinical treatments (n = 454)**	**Public health burden (n = 8)**	**Others (n = 5)**	**Others (n = 6)**	
• Hydroxychloroquine	• Seroprevalence			
• Remdesivir	• Mortality			
• Tocilizumab	**Prevention and control measures (n = 109)**			
• Cellar therapy	• Face masks			
• Traditional Chinese medicine	• Hand washing			
**Clinical trials (n = 15)**	• Social distancing			
• Vaccines	**Living habits (n = 28)**			
• Drugs	• Smoking			
**Clinical outcomes (n = 123)**	• Physical activity			
• Discharge rate, and fatality rate	**Environment (n = 7)**			
• Recurrence	**Others (n = 38)**			
• Critical complications				
**Clinical risk prediction models (n = 15)**				
• Model development and validation				
**COVID-19 and comorbidities (n = 480)**				
• Liver and kidney Injury				
• Diabetes mellitus				
• Cardiovascular diseases				
• Mental health				
**Others (n = 149)**				

**Clinical management of COVID-19 and pandemic-related health issues**, a total of 1755 reviews were divided into seven subgroups: Clinical features (n = 372); Clinical tests (n = 147); Clinical treatments (n = 454); Clinical trials (n = 15); Clinical outcomes (n = 123); Clinical risk prediction models (n = 15); and COVID-19 and comorbidities (n = 480).

**Public-health measures**, a total of 342 reviews were divided into six subgroups: Infection (n = 37); Transmission (n = 115); Public health burden (n = 8); Prevention and control measures (n = 109); Living habits (n = 28); Environmental impact (n = 7).

**Health-system arrangements**, a total of 85 reviews were divided into two subgroups: Health care resource arrangement (n = 38); Clinical department arrangement (n = 42).

**Economic and social responses**, a total of 59 reviews were divided into four subgroups: Education (n = 11); Social consequences (n = 29); Economy (n = 7); Governance (n = 6).

**Other reviews**, a total of 93 reviews were divided into three subgroups: Comparison of COVID-19, SARS, and MERS (n = 32); Publications (n = 25); Coronavirus genomic RNA packaging (n = 19).

**Figure:**
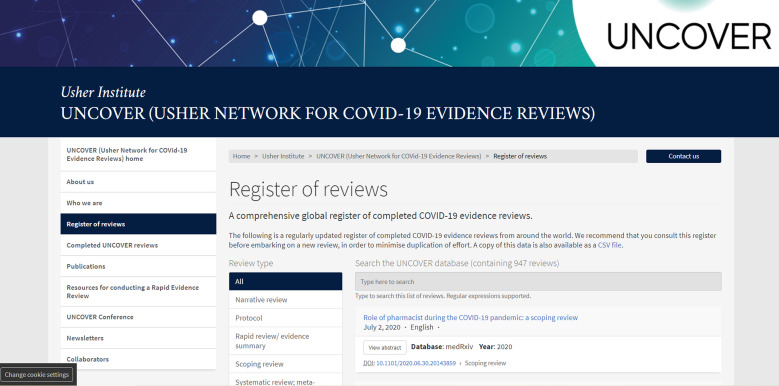
Photo: Register of reviews from UNCOVER team, the University of Edinburgh (from the author’s own collection, used with permission).

On average, we include 292 reviews per month. There has been an increasing trend in the number of published or ongoing COVID-19 reviews and the composition of research topics has changed over time. **Figure 2**, Panel B, shows that the proportion of reviews on clinical management of COVID-19 and pandemic-related health issues increased from April to September (from 60.3% to 82.3%) but the proportion has been steadily declining since September to 74.4% and 72.4% for October and November respectively. In contrast, the proportion of reviews on public health measures decreased from May to September except for August (from 21.4% to 10.5%) and the proportion was higher in October and November (16.8% and 13.9% respectively). For the other three topics, the trend is fluctuating. We further categorized the pandemic into three time periods: 1) before June 2020, where the main contributors were Western Pacific, Americas and Europe but the case incidence was kept in relatively low levels; 2) June-September 2020, where the number of new cases continued to increase and the main contributors were Americas and South-East Asia; and 3) October 2020 to now, where the number of cases increased significantly driven by the second outbreak in Americas and Europe. From **Figure 2**, Panel C, the highest proportion of reviews on clinical management of COVID-19 and pandemic-related health issues was in June to September while the proportion of reviews on public health measures was relatively high in periods before June and after September. The proportion of reviews on economic and social responses has been increasing, but they still account for a small proportion.

In summary, a total of 2334 COVID-19 evidence-based reviews have been indexed in UNCOVER registry, more than half of the reviews included were from PubMed, and over two-thirds of the included reviews were systematic reviews and meta-analyses. The research topics concentrated on clinical features, tests, treatment and outcomes, COVID-19 comorbidities, and the transmission, prevention and control of COVID-19. Although we have seen an increasing trend for reviews on economic and social responses across the three time periods, the total number remains very low and not in line with its importance.

The registry offers the opportunity to explore the aforementioned topics using state of the art methodologies in evidence-based research (such as umbrella reviews with evidence synthesis and assessment of the risk of bias) [8-12]. This will provide policymakers, clinicians and researchers a clear understanding of broad topic areas in relation to COVID-19.
